# Supplementary material for: Synergy between BRD9- and IKZF3-Targeting as a Therapeutic Strategy for Multiple Myeloma
Source: Cancers (Basel). 2024 Mar 28;16(7):1319. doi: 10.3390/cancers16071319 (PMC11010819; doi:10.3390/cancers16071319)
Supplement: Supplementary file 1 [file cancers-16-01319-s001.zip › cancers-2943949-supplementary.pdf]

Supplementary Figure 1

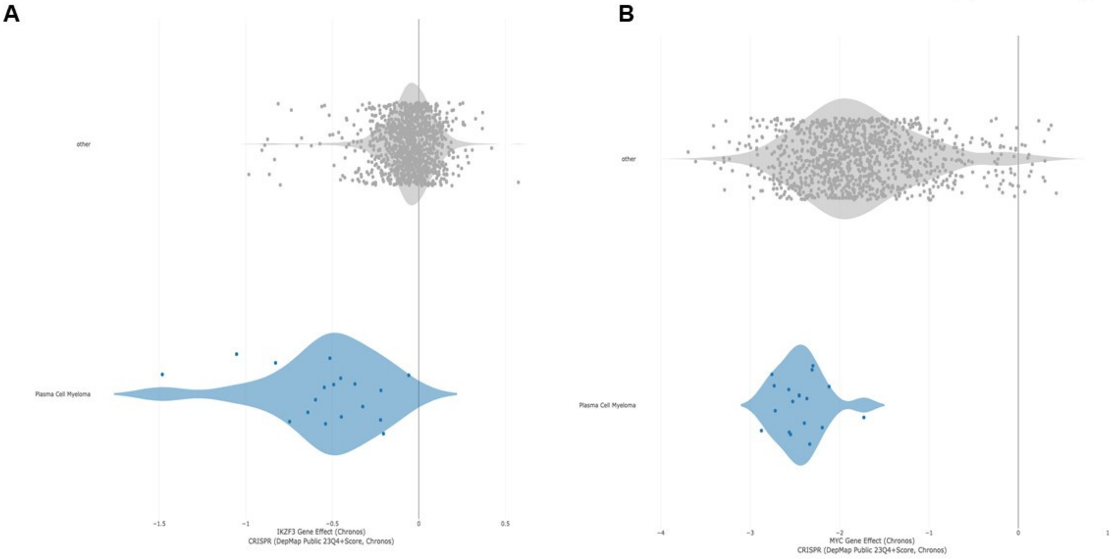

Supplementary Figure S1. Genome-based CRISPR gene-editing screen identifies preferential dependencies in myeloma cell lines. Gene effect of IKZF3 and MYC targeting in 18 myeloma cell lines (blue) versus various other cancer cell lines (gray) tested within this screen. Data were retrieved from https://depmap.org/.

Supplementary Figure 2

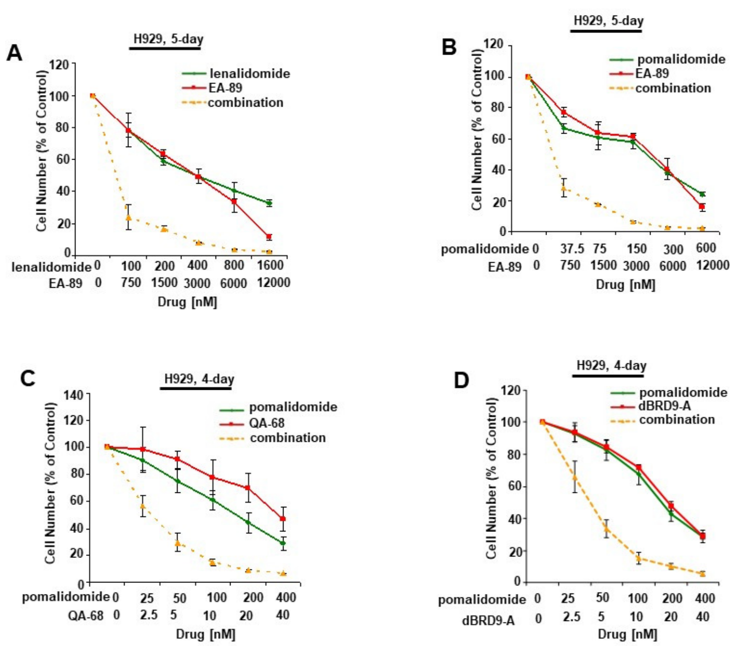

Supplementary Figure S2. BRD9 inhibitor or degrader treatment potentiates effects of IMiDs against growth of MM cells. (A-D) Proliferation assays: Effects of lenalidomide or pomalidomide alone or combined with the BRD9 inhibitor, EA-89, or the BRD9 degraders, QA-68 or dBRD9A, against H929 cells
following treatment for the indicated times. CIs for (A): ED25: 0.18, ED50: 0.16, ED75: 0.15, ED90: 0.16. CIs for (B): ED25: 0.31, ED50: 0.20, ED75: 0.14, ED90: 0.11. CIs for (C):

ED25: 0.20, ED50:0.23, ED75: 0.27, ED90: 0.32. CIs for (D): ED25: 0.34, ED50: 0.35, ED75: 0.37, ED90: 0.39.

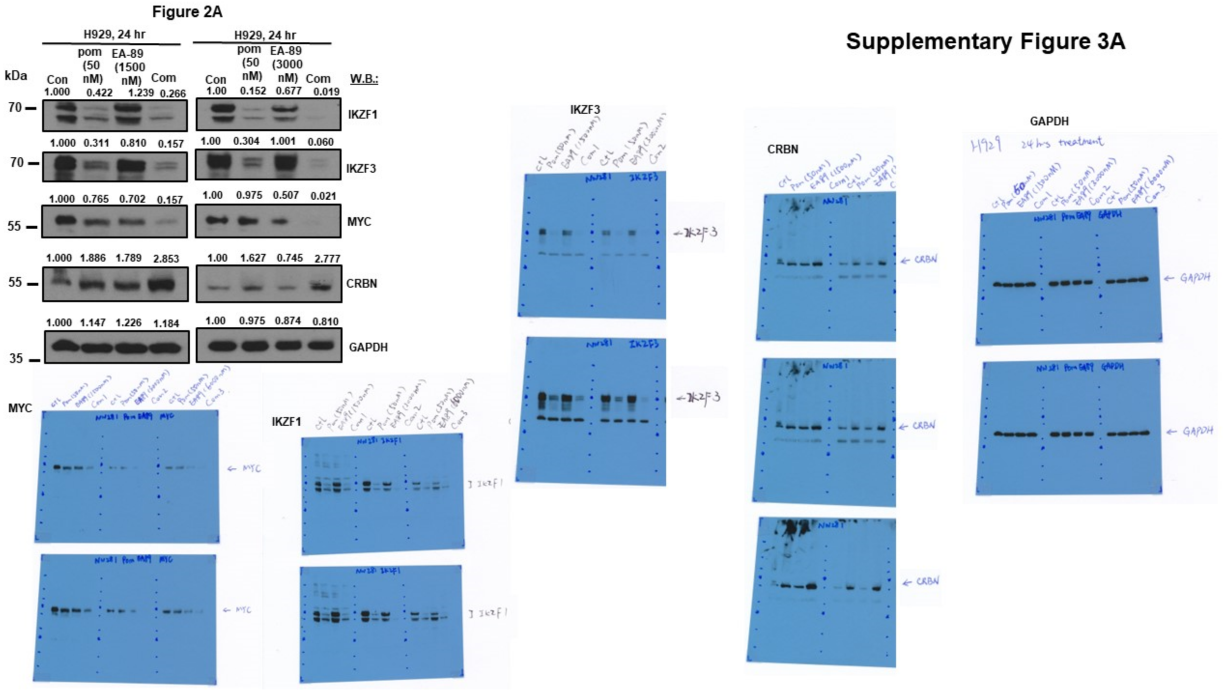

Supplementary Figure S3A: Full (uncut) immunoblots and densitometry corresponding to Figure 2A. ImageJ package was used to analyze the densitometric values (raw reads) for Western blotting bands. For the normalization results, each blot was normalized to the corresponding, far left blot (usually the control) in each group.

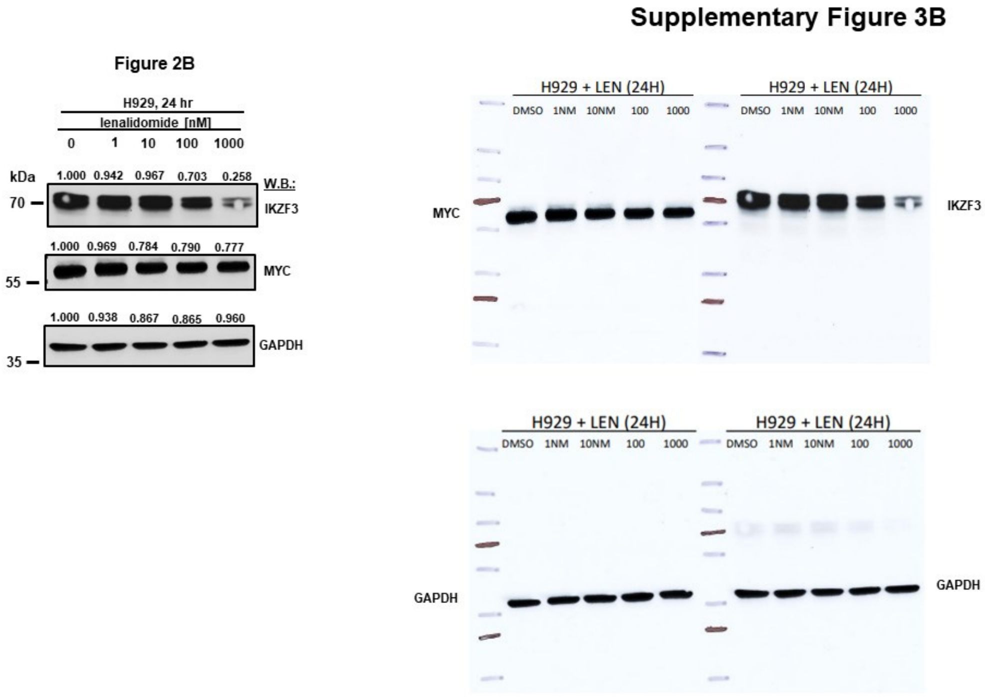

Supplementary Figure S3B: Full (uncut) immunoblots and densitometry corresponding to Figure 2B. ImageJ package was used to analyze the densitometric values (raw reads) for Western blotting

bands. For the normalization results, each blot was normalized to the corresponding, far left blot (usually the control) in each group.

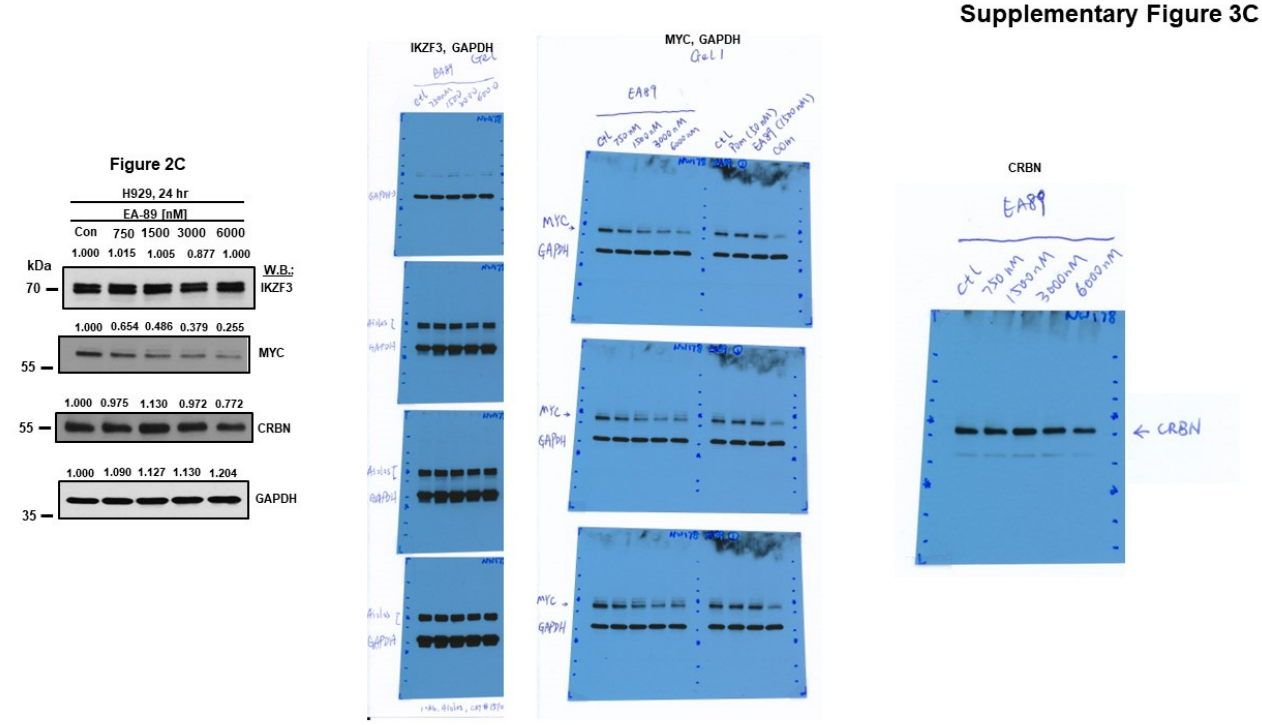

Supplementary Figure S3C: Full (uncut) immunoblots and densitometry corresponding to Figure 2C. ImageJ package was used to analyze the densitometric values (raw reads) for Western blotting bands. For the normalization results, each blot was normalized to the corresponding, far left blot (usually the control) in each group.

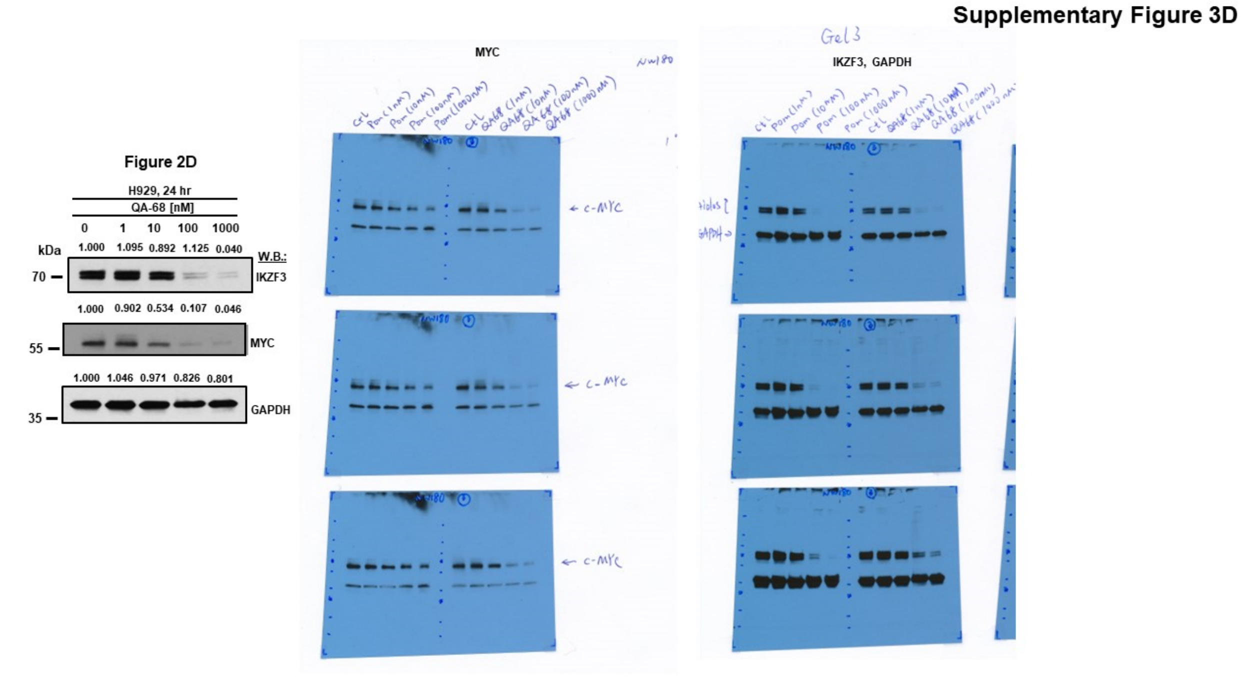

Supplemental Figure S3D: Full (uncut) immunoblots and densitometry corresponding to Figure 2D. ImageJ package was used to analyze the densitometric values (raw reads) for Western blotting

bands. For the normalization results, each blot was normalized to the corresponding, far left blot (usually the control) in each group.

Supplementary Figure 3E

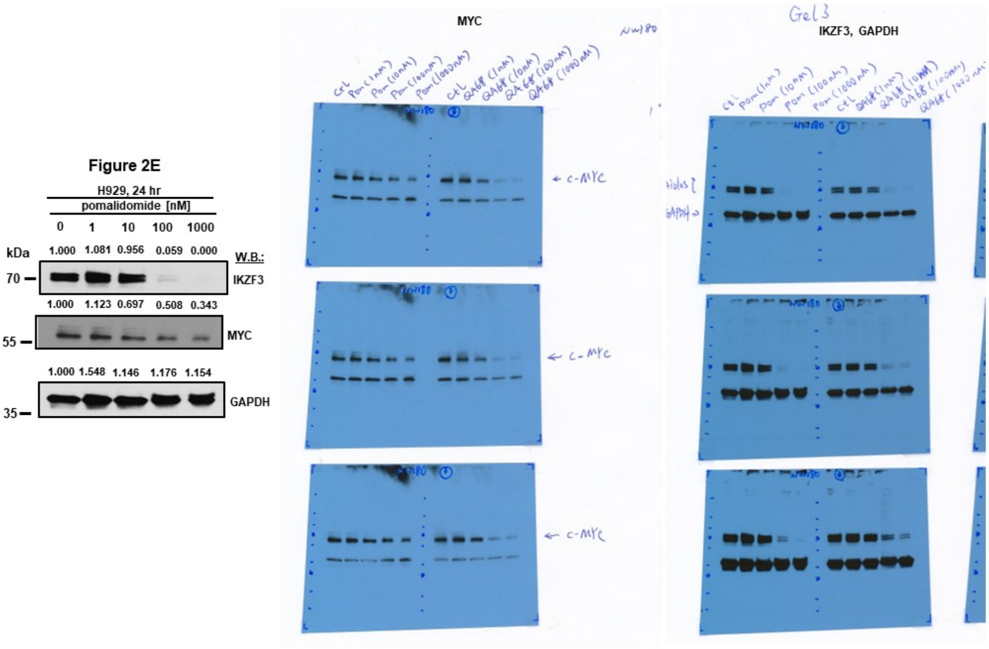

Supplemental Figure S3E: Full (uncut) immunoblots and densitometry corresponding to Figure 2E. ImageJ package was used to analyze the densitometric values (raw reads) for Western blotting bands. For the normalization results, each blot was normalized to the corresponding, far left blot (usually the control) in each group.

Supplementary Figure 4(A-E)

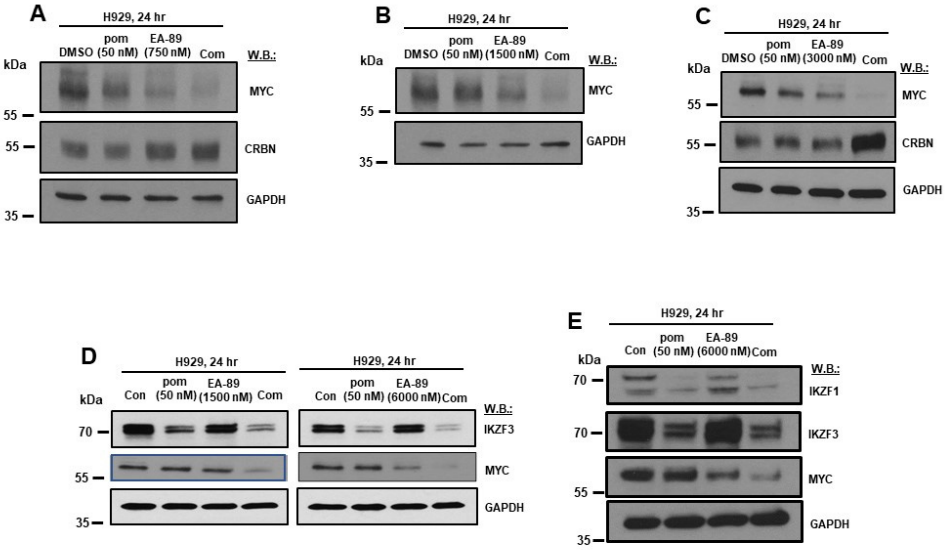

Supplementary Figure S4(A-E). Effects of pomalidomide or EA-89 alone or combined on IKZF3 and MYC protein levels in H929 cells. (A-C) Immunoblots: Effects of pomalidomide or EA-89 alone or combined on MYC and CRBN protein levels following 24 hours. (D-E) Immunoblots: Effects of pomalidomide or EA-89 alone or combined on IKZF1, IKZF3, and MYC protein levels

following 24 hours. MYC antibody: CST #5605, rabbit 1:1000 in 5% milk, IKZF1 antibody: CST#14859, rabbit 1:1000 in 5% milk, IKZF3 antibody: CST#15103, rabbit 1:1000 in 5% milk.

Supplementary Figure 4F

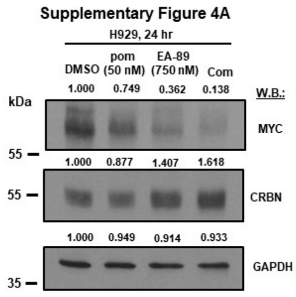

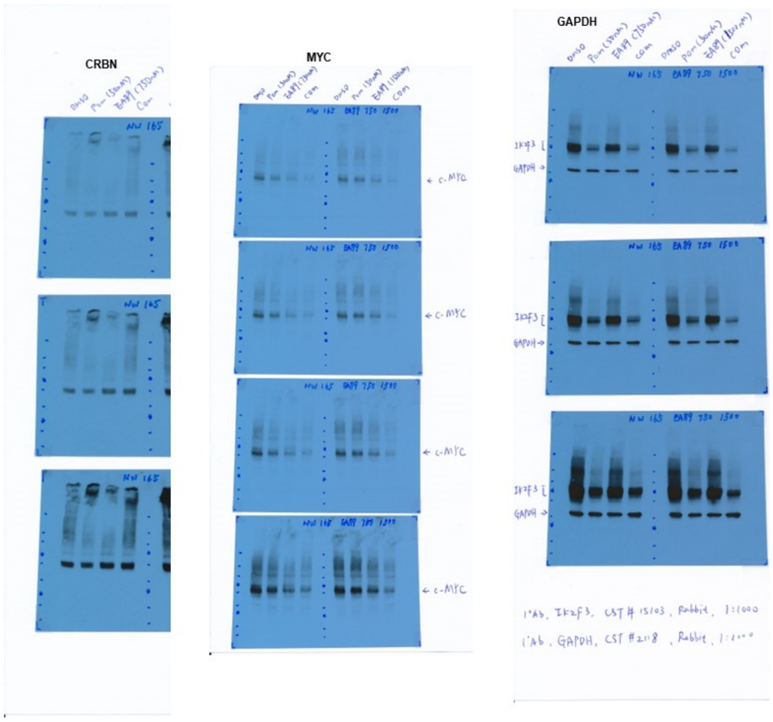

Supplemental Figure S4F: Full (uncut) immunoblots and densitometry corresponding to Supplementary Figure S4A.

ImageJ package was used to analyze the densitometric values (raw reads) for Western blotting bands. For the
normalization results, each blot was normalized to the corresponding, far left blot (usually the control) in each group.

Supplementary Figure 4G

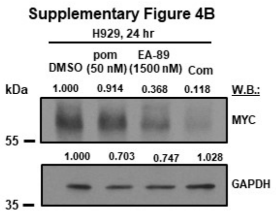

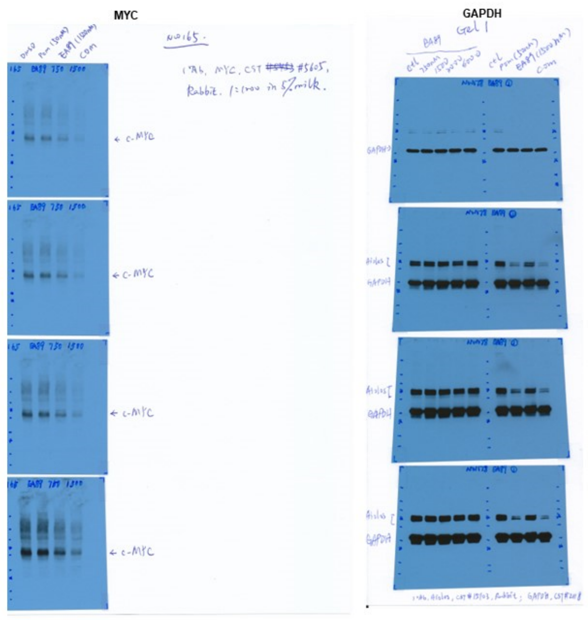

Supplemental Figure S4G: Full (uncut) immunoblots and densitometry corresponding to Supplementary Figure S4B.

ImageJ package was used to analyze the densitometric values (raw reads) for Western blotting bands. For the
normalization results, each blot was normalized to the corresponding, far left blot (usually the control) in each group.

Supplementary Figure 4H

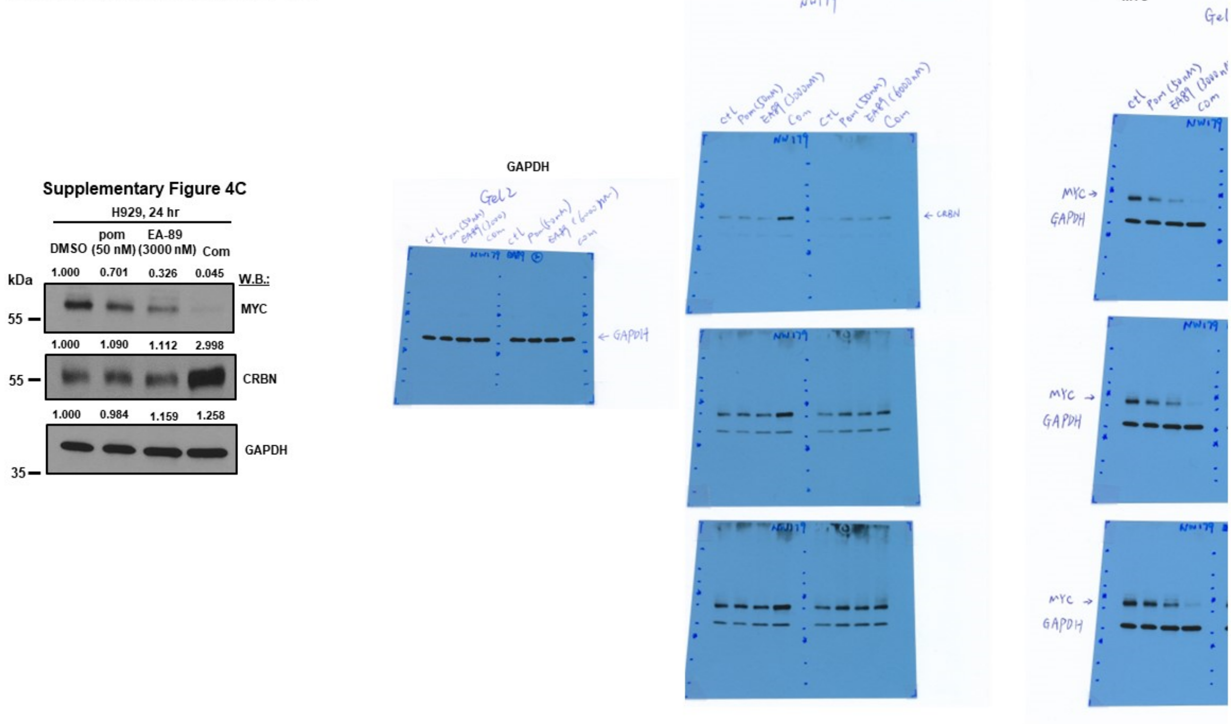

Supplemental Figure S4H: Full (uncut) immunoblots and densitometry corresponding to Supplementary Figure S4C.
ImageJ package was used to analyze the densitometric values (raw reads) for Western blotting bands. For the
normalization results, each blot was normalized to the corresponding, far left blot (usually the control) in each group.

Supplementary Figure 4I

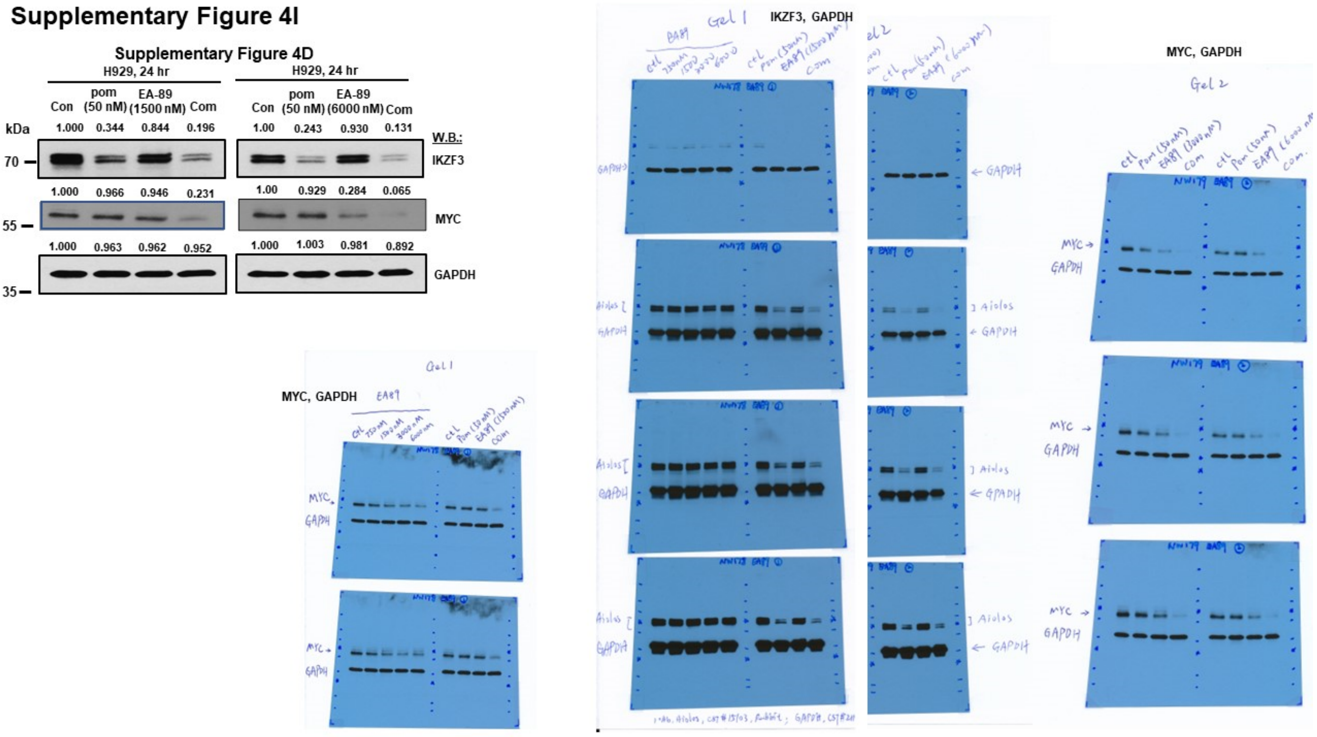

Supplemental Figure S4I: Full (uncut) immunoblots and densitometry corresponding to Supplementary Figure S4D.
ImageJ package was used to analyze the densitometric values (raw reads) for Western blotting bands. For the
normalization results, each blot was normalized to the corresponding, far left blot (usually the control) in each group.

Supplementary Figure 4J

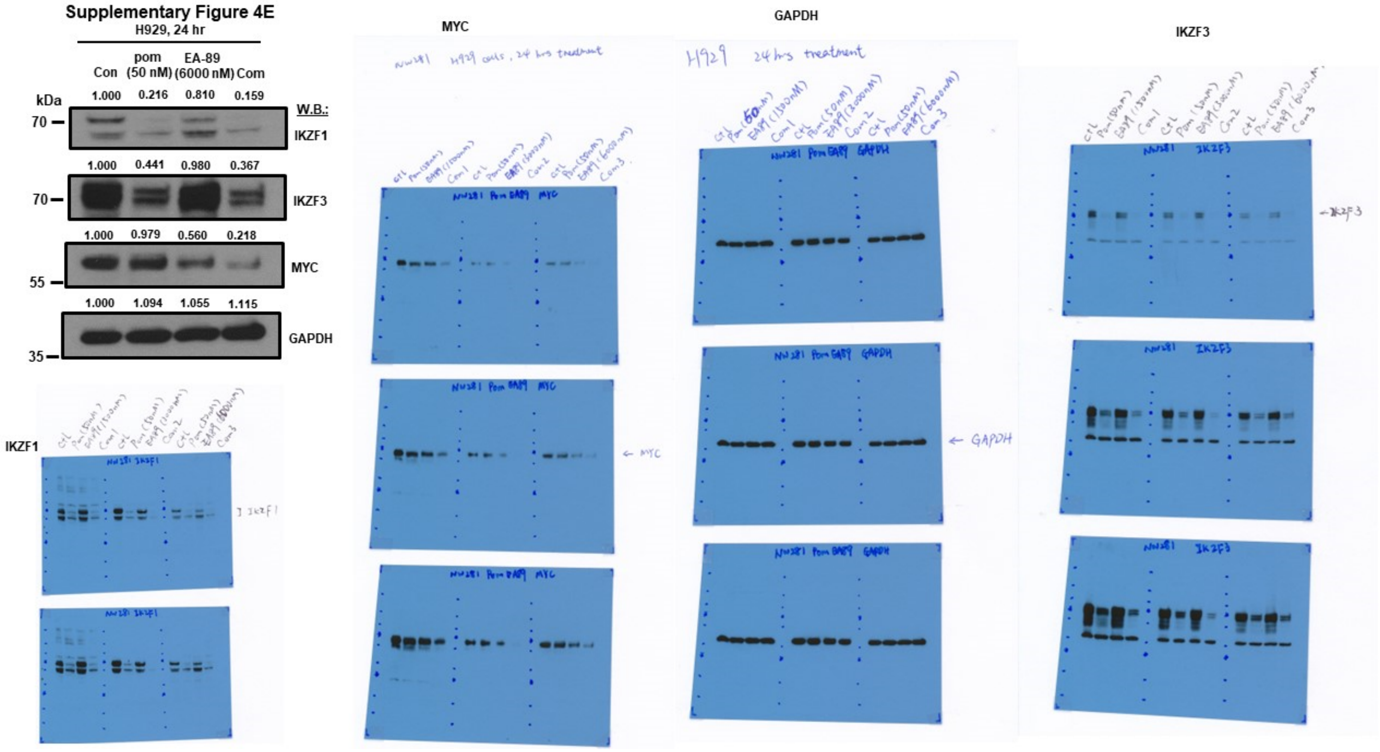

Supplemental Figure S4J: Full (uncut) immunoblots and densitometry corresponding to Supplementary Figure S4E.
ImageJ package was used to analyze the densitometric values (raw reads) for Western blotting bands. For the
normalization results, each blot was normalized to the corresponding, far left blot (usually the control) in each group.

Supplementary Figure 5

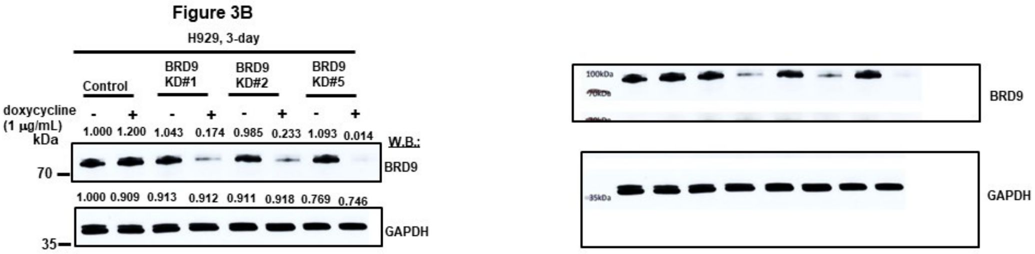

Supplemental Figure S5: Full (uncut) immunoblots and densitometry corresponding to Figure 3B. ImageJ package was used to analyze the densitometric values (raw reads) for Western blotting bands. For the normalization results, each blot was normalized to the corresponding, far left blot (usually the control) in each group.

Supplementary Figure 6 (A)

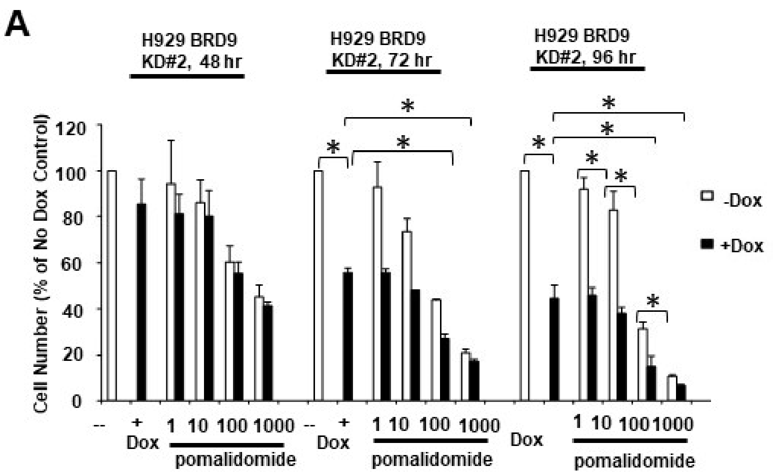

Supplementary Figure S6(A). BRD9 KD potentiates effects of pomalidomide against growth of MM cells. For H929 KD#2, pomalidomide vs pomalidomide+doxycycline, asterisks indicate p<0.05 for all pomalidomide concentrations. For H929 KD#2 and H929 KD#5, doxycycline vs pomalidomide+doxycycline, asterisks indicate p<0.02 for all pomalidomide concentrations.

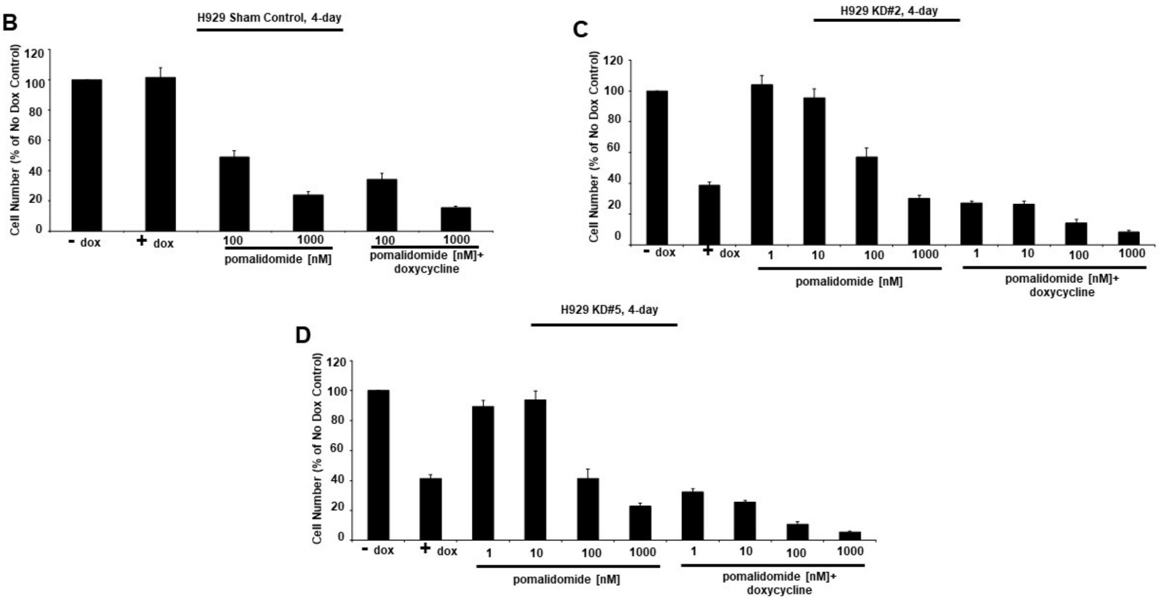

Supplementary Figure 6 (B-D)

Supplementary Figure S6(B-D). BRD9 KD potentiates effects of pomalidomide against growth of MM cells. Biological replicate (day 4 time point). For H929 KD#2 and H929 KD#5, pomalidomide vs pomalidomide+doxycycline, p<0.02 for all pomalidomide concentrations. For H929 KD#2 and H929

KD#5, doxycycline vs pomalidomide+doxycycline, p<0.02 for all pomalidomide concentrations. Statistics were derived from 3-4 technical replicates.

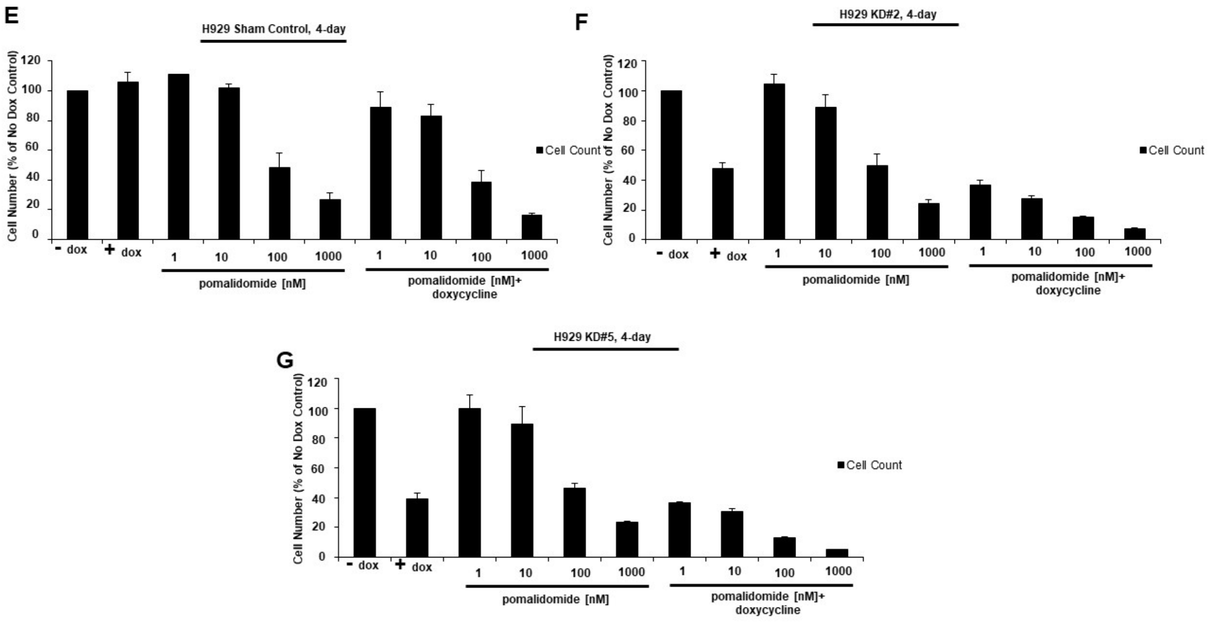

Supplementary Figure 6 (E-G)

Supplementary Figure S6(E-G). BRD9 KD potentiates effects of pomalidomide against growth of MM cells. Biological replicate (day 4 time point). For H929 KD#2 and H929 KD#5, doxycycline vs pomalidomide+doxycycline, p<0.02 for all pomalidomide concentrations, with the exception of H929 BRD9 KD#2, for which the doxycycline vs pomalidomide ((1 nM)+doxycycline is not significant. Statistics were derived from 3-4 technical replicates.

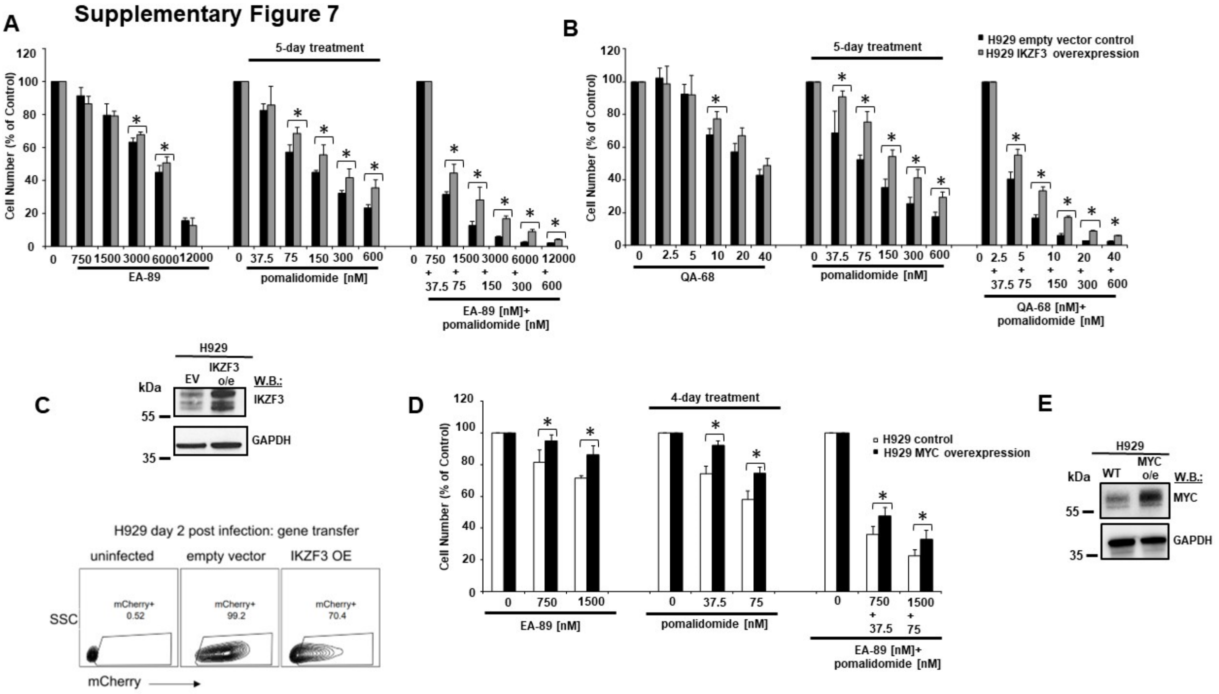

Supplementary Figure S7. IKZF3 or MYC overexpression in H929 cells partially reverses synergy between BRD9-targeted agents and pomalidomide. (A) Proliferation assay: Growth effects EA-89,

pomalidomide, or EA-89+pomalidomide treatment of H929 empty vector control cells versus H929 IKZF3 overexpression cells following 5 days. (B) Proliferation assay: Growth effects of QA-68, pomalidomide, or QA-68+pomalidomide treatment of H929 empty vector control cells versus H929 IKZF3 overexpression cells following 5 days. (C, upper panel) Immunoblot: Measurement of IKZF3 in empty vector and IKZF3 overexpression H929 cells. (C, lower panel) Flow cytometry measurement of expression of empty vector or IKZF3 overexpressed in H929 cells, 2 days post-infection. (D) Proliferation assay: Growth effects of EA-89, pomalidomide, or EA-89+pomalidomide treatment of H929 wt control cells or H929 MYC overexpression cells following 4 days. (E) Immunoblot: Measurement of MYC expression in H929 wt control cells or H929 MYC overexpression cells. For (A), (B) and (D), asterisks indicate statistical significance (p value < 0.05). Statistics were derived from at least 3-4 technical replicates.

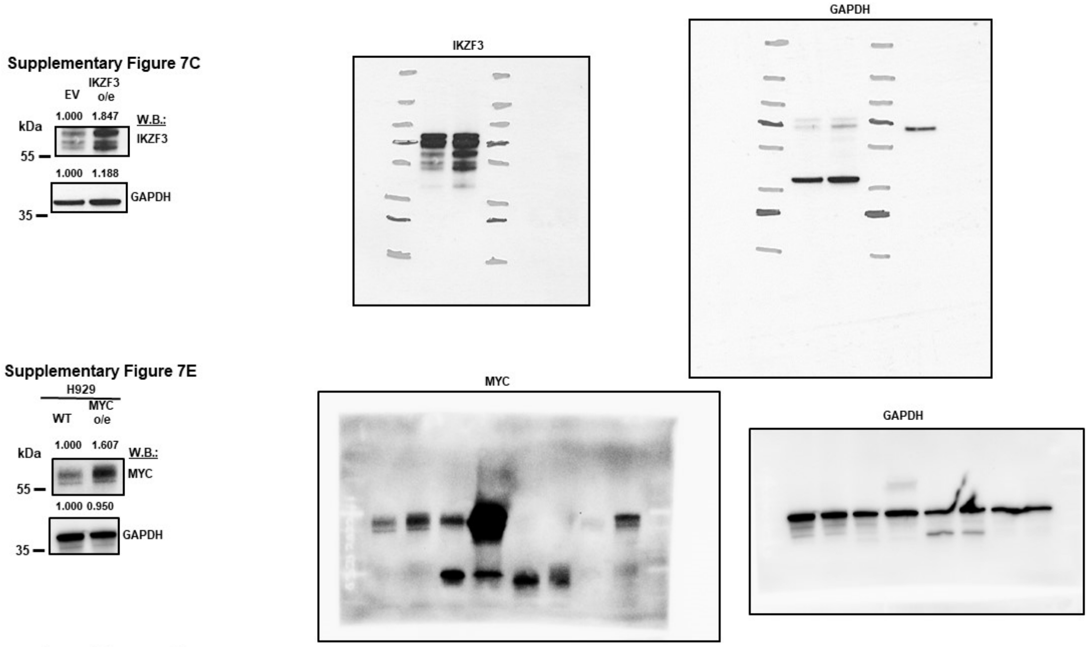

Supplementary Figure 8

Supplemental Figure S8: Full (uncut) immunoblots and densitometry corresponding to Supplementary Figures 7C and 7E. ImageJ package was used to analyze the densitometric values (raw reads) for Western blotting bands. For the normalization results, each blot was normalized to the corresponding, far left blot (usually the control) in each group.

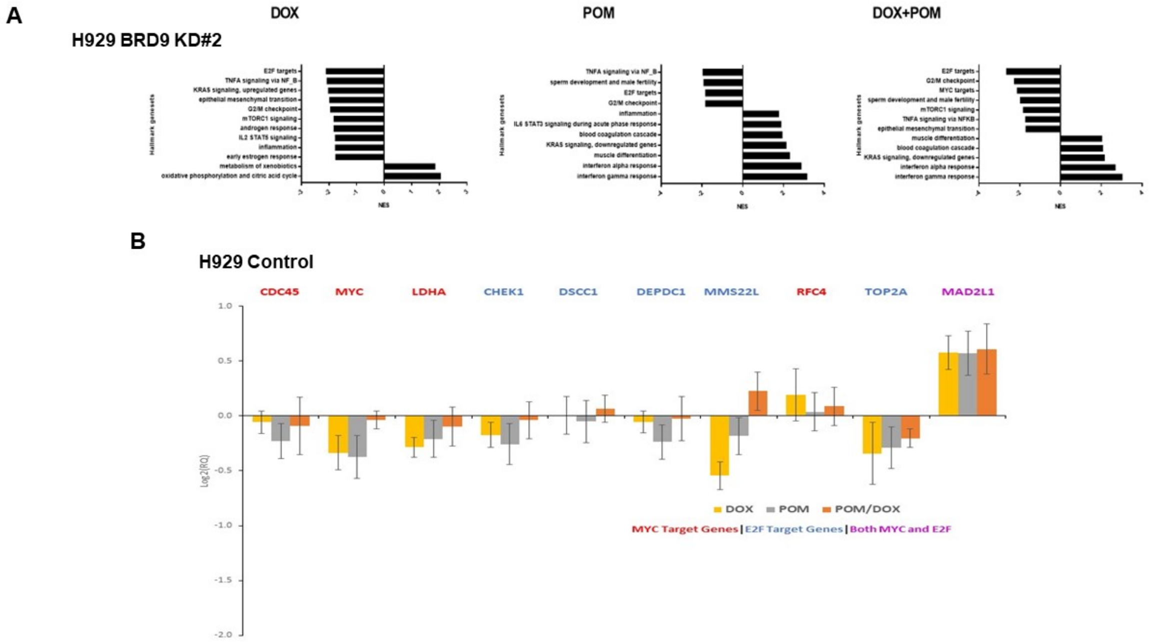

Supplementary Figure 9

Supplementary Figure S9. GSEA enrichment analysis and PCR validation of MYC and E2F target genes. (A) GSEA enrichment analysis of RNA-seq results for H929 BRD9 KD cells treated with doxycycline, pomalidomide, or a
combination. (B) PCR validation of selected MYC and E2F target genes in H929 control cells.

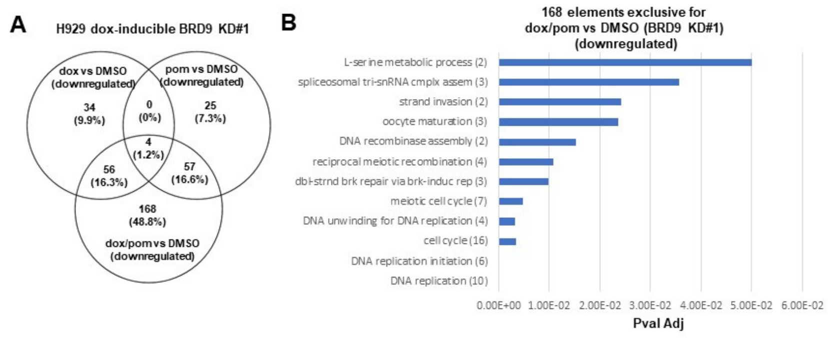

Supplementary Figure 10 (A-B)

Supplementary Figure S10 (A-B). Targeted loss of BRD9 combined with pomalidomide leads to suppression of
major signaling pathways in MM. (A) Venn diagram. (B) Pathway analysis showing effects of doxycycline-induced BRD9 KD+pomalidomide treatment of H929 dox-inducible BRD9 KD#1 cells for 24 hours. The cutoff for significance for pathways shown in (B) was 5E-02. For (B), gene numbers are shown in parentheses. Treatments were as follows: DMSO vehicle, doxycycline (1 ug/mL), pomalidomide (50 nM), and combination of doxycycline (1 ug/mL)+pomalidomide (50 nM). GeneCodis was utilized for pathway analysis, using GO Biological Process. Among the 4

genes common among dox only-, pom only-, and dox+pom-treated H929 dox-inducible BRD9 KD#1 cells are EGR2, INHBE, TNC, and CHAC1.

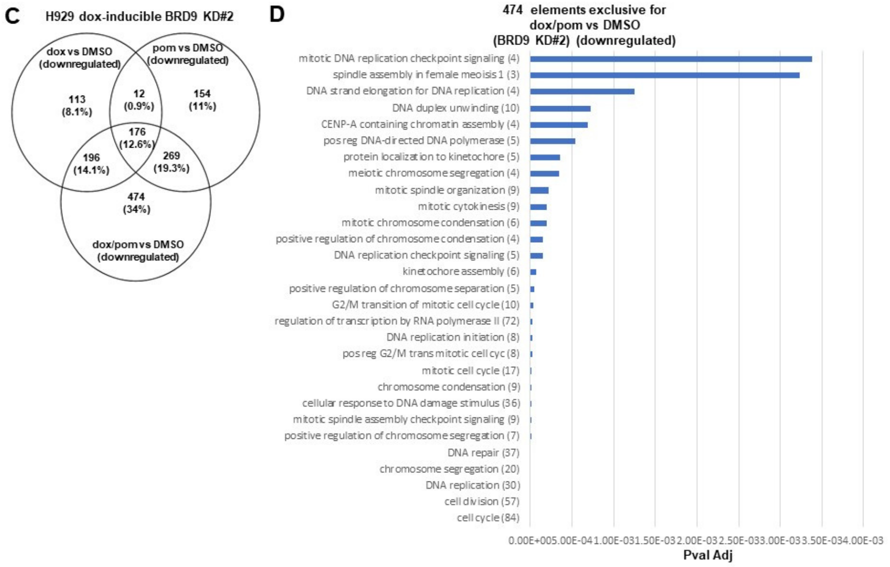

Supplementary Figure 10 (C-D)

Supplementary Figure S10 (C-D). Targeted loss of BRD9 combined with pomalidomide leads to suppression of major signaling pathways in MM. (C) Venn diagram. (D) Pathway analysis showing effects of doxycycline-induced BRD9 KD+pomalidomide treatment of H929 dox-inducible BRD9 KD#2 cells for 24 hours. The cutoff for significance for
pathways shown in (D) was P=4e-03. For (D), gene numbers are shown in parentheses. Treatments were as follows: DMSO vehicle, doxycycline (1 ug/mL), pomalidomide (50 nM), and combination of doxycycline (1 ug/mL)+pomalidomide (50 nM). GeneCodis was utilized for pathway analysis, using GO Biological Process. Among the 176 genes common among dox only-, pom only-, and dox+pom-treated H929 dox-inducible BRD9 KD#1 cells are EGR1, EGR2, and INHBE.

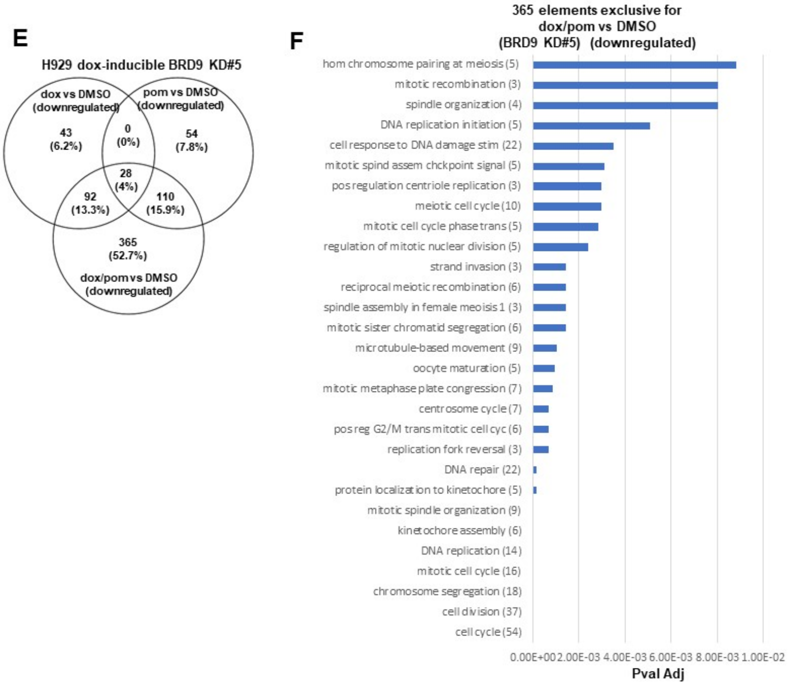

Supplementary Figure 10 (E-F)

Supplementary Figure S10 (E-F). Targeted loss of BRD9 combined with pomalidomide leads to suppression of
major signaling pathways in MM. (E) Venn diagram. (F) Pathway analysis showing effects of doxycycline-induced BRD9 KD+pomalidomide treatment of H929 dox-inducible BRD9 KD#2 cells for 24 hours. The cutoff for significance for pathways shown in (B) was 1e-02. For (F), gene numbers are shown in parentheses. Treatments were as follows: DMSO vehicle, doxycycline (1 ug/mL), pomalidomide (50 nM), and combination of doxycycline (1 ug/mL)+pomalidomide (50 nM). GeneCodis was utilized for pathway analysis, using GO Biological Process. Among the 28 genes common among dox only-, pom only-, and dox+pom-treated H929 dox-inducible BRD9 KD#5 cells are INHBE, EGR1, GABRG1, OLAH, SLC12A8, SLC7A11, MKX, LOC105278047, SRGN, NPR3, TNC, CTH, LOC730100, LOC101928932, PDE4D, GALNT3, PHLDA1, CD55, RPL23AP7, FAM114A1, B4GALT6, RNU5A-1, LOC613266, MB, ZNF519, NUP62CL, SBF2-AS1, and TMEM56.

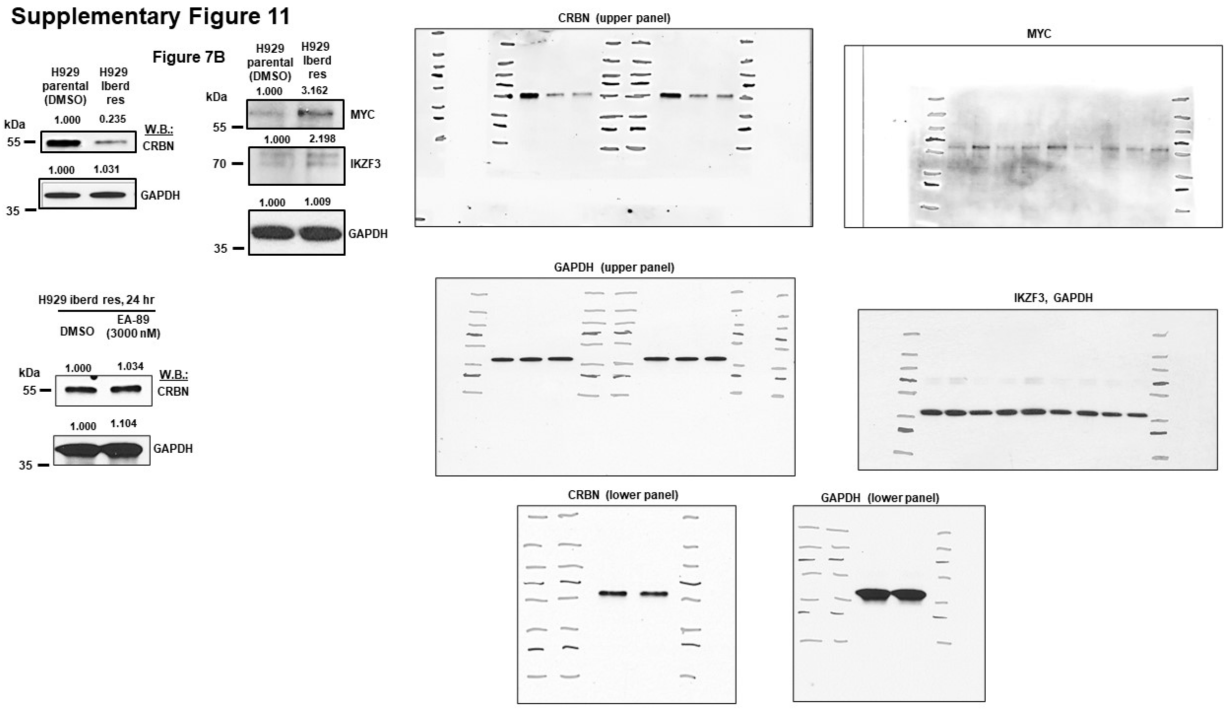

Supplemental Figure S11: Full (uncut) immunoblots and densitometry corresponding to Figure 7B. ImageJ package was used to analyze the densitometric values (raw reads) for Western blotting bands. For the normalization results, each blot was normalized to the corresponding, far left blot (usually the control) in each group.

Supplementary Figure 12

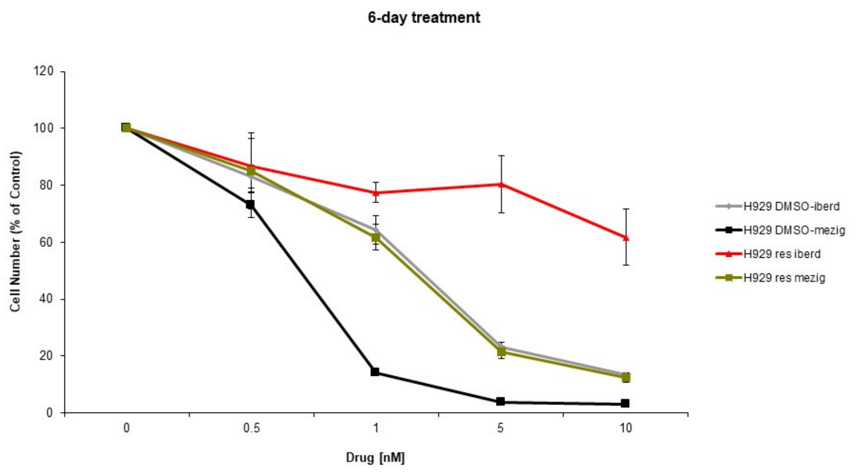

Supplementary Figure S12. Proliferation assay: Effects of iberdomide or mezigdomide against parental H929 cells (treated with DMSO for four months) or IMiD-resistant H929 cell line#2 (treated for four months with 10-20 nM
iberdomide following 6 days.
